# Single-Use Flexible Ureteroscopes: How Difficult Is It Today to Stay Up to Date? A Pictorial Review of Instruments Available in Europe in 2023

**DOI:** 10.3390/jcm12247648

**Published:** 2023-12-13

**Authors:** Chiara Vaccaro, Vito Lorusso, Franco Palmisano, Marco Rosso, Massimiliano Nicola, Antonio Maria Granata, Andrea Gregori, Michele Talso

**Affiliations:** 1 Department of Urology, ASST Fatebenefratelli Sacco, 20157 Milan, Italy; chiara.vaccaro@unimi.it (C.V.);; 2 University of Milan, 20122 Milan, Italy

**Keywords:** disposable ureteroscopes, single-use, RIRS, urolithiasis

## Abstract

The invention of the flexible ureteroscope (fURS) and its subsequent spread have revolutionized the surgical management of urolithiasis and upper tract urothelial carcinoma (UTUC). During the last few years, single-use flexible ureteroscopes (su-fURSs) have been developed to improve the limitations of reusable fURSs, namely their cost, durability and risk of device contamination. Since the introduction of the first fully disposable digital fURS, several su-fURSs have been developed by various manufacturers. In this pictorial review, we combined the different physical and technical features of su-fURSs currently available on the market with Food and Drug Administration (FDA) and European Conformity (CE) approval, in order to help surgeons choose the appropriate device according to each case requirement and personal preferences. To the best of our knowledge, 17 su-fURSs with CE and FDA approval have been developed to date.

## 1. Introduction

The invention of the flexible ureteroscope (fURS), firstly reported by Marshall in 1964, and its subsequent spread have revolutionized the management of various urological conditions [1]. Nowadays, according to European Association of Urology (EAU) guidelines on urolithiasis and upper tract urothelial carcinoma (UTUC), the fURS represents the first-line treatment option for stone disease of the kidney and for the conservative management of UTUC [2,3]. However, its high initial and maintenance costs due to repairs and sterilization and its limited durability can represent some of the limits of these surgical instruments [4,5,6]. Moreover, the risk of device contamination and nosocomial infection has been widely reported [4,7]. To improve these limitations, single-use flexible ureteroscopes (su-fURSs) have been developed during the last few years.

Since the introduction of the first su-fURS, several devices have been developed and, nowadays, a large number of su-fURSs are available on the market.

These devices differ in some technical characteristics, including weight, diameter (outer, tip and working channel), deflection, irrigation and image resolution. Each of these features may represent a pro or a con depending on the situation and personal preferences. The aim of this pictorial review is to analyze the different features of disposable devices currently available on the market that are both Food and Drug Administration (FDA)- and European Conformity (CE)-certified to the best of our knowledge.

## 2. Development of Disposable Ureteroscopes

The first transurethral flexible ureteroscope was reported by Marshall in 1964. This early flexible ureteroscope was limited in its capabilities as it did not have working channels and active deflection [1,8]. Since the first ureteroscope (URS) was introduced, the field of ureterorenoscopy has undergone continuous evolution as a result of the technological advances that have occurred during the last few decades, including the introduction of disposable URSs.

The first disposable URS was produced by VanTec in 1985. It was composed of a disposable flexible shaft with a non-deflectable tip of 7 and 8.5 Ch and a reusable handle containing the optics [8]. Bard developed a similar disposable URS, which consisted of a disposable flexible shaft with a deflectable tip and reusable handle. They were both fiber-optic devices. In the following years, other semi-disposable ureteroscopes were introduced on the market, namely SemiFlex in 2009, Polyscope in 2011 and FlexorVue in 2013.

All these devices consisted of a disposable flexible shaft with a deflectable tip and a reusable fiber-optic bundle. The poor deflecting mechanism of these devices limited their spread [8,9]. However, these devices have opened up the path to the next generation of URSs. 

In recent decades, different companies have developed various fURS models characterized by complete deflection capabilities. Since an adequate quality of vision is mandatory if surgeons want to achieve better outcomes, flexible scopes offer improved vision based on the incorporation of an increased number of fiber-optic bundles or the integration of a distal sensor chip [10,11].

Until the twenty-first century, fiber optics remained the sole optical system for visualization. However, the introduction of the first digital URS marked a significant milestone in the advancement of flexible ureteroscope technology [8]. The introduction of digital URSs has offered many advantages for diagnostic and treatment procedures, with a significant reduction in operative times when treating stones due to the substantial improvement in image quality [12]. Indeed, in terms of image resolution, both reusable and disposable digital fURSs outperform the fiber-optic models [13]. LithoVue was the first fully disposable digital fURS developed by Boston Scientific in 2015 and approved by the FDA [14,15]. Since its introduction, several su-fURSs have been developed by various manufacturers in Europe, North America and Asia.

## 3. Disposable Flexible Ureteroscope Features

To select the scopes to include in this paper, we conducted an extensive literature search to evaluate all the devices currently available on the market with CE and FDA approval. Moreover, we looked at all the booths present at the EAU annual congress 2023 in Milan, selecting the ones presenting a single-use fURS to further assess all the features of the devices.

We collected 17 su-fURSs with CE and FDA approval to date.

Different physical and technical features of the currently available digital URSs are detailed in Table 1, focusing on size (outer, tip and working channel diameters), irrigation, deflection and image resolution.

### 3.1. Size and Weight

The size and weight of ureteroscopes represent some of the most important characteristics of disposable devices. An adequate size, both of the shaft and end tip, is an important feature to have to provide easy access to the ureteral orifice, avoid ureteral lesions and allow the insertion of the scope in the ureteral access sheaths (UASs) [9,14].

As shown in Table 1, these characteristics may different significantly.

The shaft length of flexible URSs may vary between 65 and 75 cm, and the outer shaft size ranges from 7.5 to 9.6 Ch. Uscope 3033A, Urofino UV-US110-H, LScope URS3006E, Axis Leo K1046369 and HU-30S have the smallest outer shaft diameter of 7.5 Ch (Table 2). 

The tip diameter ranges from 6.6 to 9.6 Ch. LScopes have the smallest tip size of 6.6 Ch (URS3006E) and 7.2 Ch (URS3016E), followed by WiScope OTU-100 with a diameter of 7.4 Ch (Table 2).

These features allow the insertion of all disposable fURSs in the UASs currently available on the market, except for Lithovue, Axis Leo K1048308 and both EU-Scopes, which cannot be used with 9.5/11.5 Ch UAS. As this consideration relies on the measurements declared by manufacturing companies, in vitro studies are required to verify the compatibility between su-fURSs and different available UASs.

Moreover, it is evident that the use of heavier devices leads to more discomfort, muscular fatigue and wrist problems among endourologists. In support of this, a survey revealed that 32% of endourologists regularly performing ureterorenoscopy reported hand and wrist problems. Lightweight disposable fURSs with ergonomic handles may represent a way to improve maneuverability and procedural comfort with minimal fatigue [16]. 

NeoFlex NFU 9 is the lightest of all su-fURSs, weighing just 119 g (including the cable), while LithoVue is the heaviest (276 g, including the cable) (Table 2) [17]. However, all disposable ureteroscopes are lighter when compared to reusable scopes, the weight of which varies from 309 to 942.5 g [16].

### 3.2. Working Channel and Irrigation

The size of the working channel is another important feature to consider. An adequate working channel size makes fURSs suitable for the insertion of accessory devices, such as guide wires, laser fibers and retrieval baskets. 

As shown in Table 1, most of the devices currently available have 3.6 Ch working channels, with the exception of FLEX-XC1, which has a working channel diameter of 3.5 Ch (Table 2). The size of the working channel also determines the irrigation flow, which is of utmost importance in removing blood and stone fragments, improving visibility during the endoscopic procedure.

The irrigation flow rate of LithoVue, Uscope 3022 and Neoflex NFU 9 has been evaluated by Dragos et al. In vitro, irrigation rates through an empty channel with saline at a 40 cm height ranging from 18 (Neoflex NFU 9) to 26 mL/min (Uscope 3022) were observed. In all su-fURSs, a decrease in the irrigation flow rate was observed with the increase in the size of the accessory devices inserted through the working channel [17]. In another study, Baghdadi et al. suggest that the insertion of laser fibers equal to or smaller than 273 μm in a 3.6 Ch working channel does not necessarily reduce the flow rates and, therefore, endoscopic vision [18].

The irrigation flow rates of Lithovue and Uscope 3022A were recently compared by Winship et al. [19]. The irrigation flow rate through an empty channel with saline at a 50 cm height was significantly higher with Uscope 3022A.

Another parameter that plays an important role in the effectiveness of the surgical procedure is the working channel position. All the current available su-fURSs have a 3 or 9 O’clock working channel positions, except WiScope OTU-100, Axis Leo and NeoFlex NFU 9, the working channel positions of which are at 12 O’clock (Table 1).

### 3.3. Deflection

The deflection capability, defined as the range of motion at the distal tip of the scope [4], is of utmost importance during flexible ureterorenoscopy to treat lower-pole stones and access difficult calyces.

All the current available su-fURSs have a bidirectional deflection of the tip controlled by a deflection lever ranging from 270 to 280° when the working channel is empty. HU-30 and HU-30S are the only models that offer a bidirectional capability of 285° (Table 2). 

The deflection capability of Lithovue and Uscope 3022A has been evaluated in an aforementioned study by Winship et al. [19]. According to the authors, Uscope 3022A demonstrated greater average deflection with an empty working channel compared to that of LithoVue (291.1° vs. 280.3). However, a greater loss in the degree of deflection with a laser fiber in the working channel was demonstrated by Uscope 3022A (15.5° vs. 8.3°). No statistical difference in deflection loss with a basket in the working channel was observed between the scopes [19]. The deflection capability of Uscope3022A was also evaluated by Agrawal et al., and compared to that of the slimmest version, Uscope3033A [20]. The deflection capability of scopes was evaluated as lower-pole accessibility with and without accessory instruments in the working channel. According to the authors, no statistically significant differences were observed between the two models.

Another feature of the disposable devices is their self-locking capability, which may be adopted to reduce muscle fatigue in long-lasting and challenging cases. FLEX-XC1, Uscope, Wiscope OTU-100, LScope and Axis Leo have this characteristic, while EU-Scope US 31D-12 and RP-U-C12 have a self-locking capability controlled by an additional lever on the handle through which deflection can be locked (Table 1). This latter additional feature can be either a pro or a con depending on each surgeon’s preferences.

### 3.4. Image Resolution

Clear visibility is a key factor in su-fURSs. Optical properties of su-fURSs that are important during endoscopic procedures include image resolution, field of view and depth of field. As shown in Table 1, most of su-fURSs have a 160 K pixel resolution, ensuring high-quality images. The field of view of different models varies between 85° and 120°. Both EU-Scopes, LScopes, Axis Leo scopes, Uscope 3033A, HU-30 and HU-30S have a wider field of view, followed by Neo-Flex NFU 9 and both Urofino URSs.

### 3.5. Other Features

The handpiece of the ureteroscopes may have buttons that can be used for imaging and video recording and for white balance. EU-Scope US 31D-12, Uscope 3033A and 3022A, LScope, Axis Leo and HU-30/30S have image capture buttons on the ventral part of the handle that are easily accessible with one hand to take pictures while operating. Moreover, unlike other su-fURSs, RP-U-C12 is characterized by a detachable cable.

## 4. Discussion

During the last few years, the surgical management of urolithiasis and UTUC has been revolutionized by the technological advantages that have been implemented. The pivotal innovation in the field of ureterorenoscopy was the introduction of digital image sensors in 2006. The advent of digital sensors that transmit a digital signal to the image processor, namely charge-coupled devices (CCDs) and complementary metal-oxide semiconductors (CMOS), made digital ureterorenoscopy possible [21]. 

As technology advanced, digital image sensors were continually miniaturized and placed on the tip of the URSs, coining the well-known phrase “chip on the tip” [12,21]. In the current landscape, almost all models utilize CMOS sensors, which require less energy, process images faster, and are more cost-effective compared to CCD sensors [21].

Up to the introduction of digital technology, an important limitation of fURSs was the durability of optical fibers. They could easily be damaged during endoscopic procedures, especially if deflected significantly, resulting in a worse quality of vision. One of the main benefits of early digital URSs was the provision of improvements in quality and image resolution compared to that of their fiber-optic counterparts [13,21,22]. The improved visibility also plays a role in clinical performance. In a study conducted by Somani et al., a digital fURS (URF-V, Olympus) was compared to a fiber-optic fURS (URF-P5, Olympus) in 118 procedures, revealing a significantly longer mean operative time with the latter [12].

Nevertheless, reusable fURSs, both digital and fiber-optic, are very fragile, and the repair costs are difficult to calculate due to differences between countries’ rules. 

Other technological advantages, such as reductions in caliber and improvements in deflection capabilities, have characterized the new generation of digital fURSs.

Another milestone in the field of flexible ureterorenoscopy was the introduction of LithoVue in 2015 by Boston Scientific [15]. The first clinical evaluation of LithoVue was conducted by Doizi et al., who evaluated its performance before and after a procedure. Image quality, determined through images taken with the scope pre- and post-procedure, active deflection, calculated by photographing the device in complete deflection in both directions, and maneuverability, assessed using a Likert scale ranging from “bad” to “very good”, were analyzed [23].

Several in vitro, in ex vivo and in vivo studies have been conducted to compare the performance of LithoVue in terms of image resolution, color reproducibility, maneuverability and deflection capabilities, with those of reusable fiber-optic and digital fURSs [13,17,24,25]. These studies have concluded that the performance of LithoVue is satisfactory and comparable with the performance of reusable digital URSs. 

Further studies have been conducted to compare the technical characteristics of other su-fURSs with those of reusable fURSs. An ex vivo study conducted by Bourdoumis et al. aimed to assess and analyze the technical features and performance of Uscope 3022 (Pusen Medical Technology Co.) with those of the reusable digital fURS [26]. Again, the performance of Uscope 3022, especially in terms of image quality, depth perception and ergonomic handling, is comparable that of reusable URSs.

Although to date there are no studies comparing all the available disposable fURSs on the market, several studies have been conducted to compare the characteristics of these devices [19,20,27,28,29]. In terms of image quality and maneuverability, Patil et al. and Winship et al. observed no significant differences between Lithovue and Uscope 3022A [19,27]. Conversely, these results do not support the previous in vitro analysis of Marchini et al., who observed higher image resolution and more comfortable handling with LithoVue compared to Uscope 3022A [28]. Uscope 3022A was also compared with its slimmest counterpart (Uscope 3033A), revealing comparable intraoperative quality of vision and deflection, both with and without accessory instruments in the working channel [20]. Other studies have recently been conducted to assess the technical features of EU-scope (Innovex AnQing Medical), which have demonstrated its comparable image resolution and maneuverability to those of LithoVue [29].

In addition to the fact, as stated, that the performance of su-fURSs can be considered equivalent or superior to that of reusable URSs, they have overcome some of the limitations of the latter.

First, reusable URSs are expensive and require a high initial investment covering the purchase of the device itself, as well as additional costs for a light source, camera, image processor, monitors and cables. It should be noted that costs also include the recurring costs related to reprocessing, as reusable URSs require hospital specialized personnel and costly cleaning products after each procedure. Moreover, the reprocessing procedure also includes time-related costs [4,30]. Second, reusable URSs have a potentially limited lifespan as they are at risk of damage during reprocessing or surgeries. Multiple factors, such as the presence of lower-pole stones, the complexity of cases because of anatomical factors (an acute infundibulum–pelvic angle, narrow infundibulum or long calyx), the sterilization method and the inexperience of surgeons and nurses may increase the chance of damage [30,31]. Significant variability in the average number of procedures before damage occurs is reported in the literature, ranging from 6 to 21 cases for fiber-optic URSs and 10 to 21 cases for digital URSs [21,30]. In addition to the inconvenience of the device’s unavailability, the repair procedure consumes both time and financial resources. Moreover, another disadvantage of reusable URSs is the potential risk of device contamination and infectious complications [4,5]. In a recent study, 12% of the cultures obtained from fURSs were found to be positive despite their high-level disinfection [7]. This finding highlights the potential value that single-use ureteroscopes may hold in preventing the transmission of infections.

The use of su-fURSs is improving all these limitations, and they have started to spread on the market.

These findings are not consistent with those of the systematic review published in 2019 on the procedural cost of resusable fURSs. According to this review, the cost per procedure performed using a reusable fURS ranges from USD 120–1212 per procedure. Therefore, up until 2019, reusable URSs seemed to be more cost-effective in high-volume centers than su-fURSs were [32]. The threshold for preferring reusable URSs over su-fURSs was 61 cases per year per institution. However, at the time the review was published, su-fURSs were new entrants on the market and only few su-fURS were present on the Europe (EU) and United States (US) markets. 

Conversely, over the past few years, many companies have started to sell their products on Western countries’ markets, resulting in a highly competitive landscape with a large number of su-fURSs available. Moreover, in the very near future, several new devices will be launched on the market. Several advances in design and in technical characteristics are expected to occur in the near future, offering the potential to improve the existing features of su-URSs. A possible future direction includes the development of smaller image sensor chips, which could lead to a potential reduction in tip diameter. This innovation could also greatly benefit pediatric cases. Other potential directions could involve achieving a wireless scope connection to monitor or integrating temperature and pression sensors into the scope’s tip, which can represent an important safety feature with which to measure and control temperatures and the intrarenal level of pression. Suction capability could also represent a significant advancement for su-fURSs. The ability to suction fluid has the potential to decrease intrarenal pressure and heat production, thereby reducing potential complications during surgical procedures. Additionally, it allows the removal of stone fragments, improving the quality of vision in a procedure without risking scope damage [9,33]. With regard to new technologies, an increasingly significant emphasis is being placed on robotic platforms for URSs. The URS robotic platform consists of a console and a manipulator, through which a robotic arm can be manipulated. However, its dissemination remains limited to date due to the high initial costs and spatial constraints it requires [33]. 

The environmental impact of su-fURSs should be taken into account. To evaluate the impact of reusable versus single-use fURSs, a study conducted by Davis et al. was performed [34]. This comparative study is, to date, the only study that has evaluated the environmental impact of reusable and su-fURSs. They compared the carbon footprint of LithoVue with that of Olympus URV-F, by evaluating all relevant aspects, i.e. manufacturing, repairs, replacement and disposal. The conclusion of this evaluation was that the environmental impacts of reusable fURSs and su-FURSs are similar (4.47 and 4.43 kg of CO2 per case, respectively) [34].

However, as su-fURSs are made up of plastic (90%), steel (4%), electronics (4%) and rubber (2%), one of the primary concerns related to su-fURSs involves the quantity of physical waste they produce [34]. As these raw materials can take hundreds of years to decompose, non-reusable devices must be properly disposed of to avoid environment contamination.

A future perspective might consider the recycling of raw materials of the scope or the use of biodegradable materials that minimize the negative impact of URSs on the planet.

## 5. Conclusions

In conclusion, su-fURSs have emerged as a promising alternative to traditional reusable URSs in the field of ureterorenoscopy. A large number of su-fURSs are nowadays available on the market, allowing a decrease in the cost of the devices per unit. These devices differ in some physical and technical characteristics, including size, weight, irrigation, deflection and image resolution, that should be taken into account when urologists have to decide what instrument to add to their armamentarium. 

While there are still some questions about their environmental impact, it is likely that su-fURSs will continue to increase in popularity and be adopted by an increasing number of surgeons. Ideally, multiple choices should be available in healthcare facilities, allowing the surgeon to choose a device in accordance with each case requirement and personal preferences, keeping all physical and technical features in mind.

## Figures and Tables

**Table 1:** Su-fURSs currently available on the market that are both FDA- and CE-approved.

	FoV *	Shaft Length (mm)	Weight (g)	Tip Diameter (Ch)	Outer Shaft Diameter (Ch)	Working Channel (Ch)	Position Working Channel (O’clock)	Deflection	Resolution (Pixel)	Depth of Field (mm)
LithoVue, Boston Scientific	85°	680	276	7.7	9.5	3.6	3	270°		2–50
Axis Leo K1046369, Dornier MedTech	120°	650	-	7.5	7.5	3.6	12	270°	400 × 400	3–50
Axis Leo K1048308, Dornier MedTech	120°	650	-	9.6	9.6	3.6	12	270°	400 × 400	3–50
HU-30S, HugeMed	120°	650	-	-	7.5	3.6	3–9	285°	400 × 400	3–100
HU-30, HugeMed	120°	650	-	-	9	3.6	3–9	285°	400 × 400	-
EU-Scope, US 31E-12 Innovex AnQing Medical	120°	670	-	9.6	8.7	3.6	9	275°	400 × 400	3–100
EU-Scope, US 31D-12 Innovex AnQing Medical	120°	670	130	9.6	8.7	3.6	9	275°	400 × 400	3–100
Neo-Flex NFU 9, Neoscope Inc.	110°	680	119	9	9	3.6	12	280°	400 × 400	5–50
WiScope OTU-100, OTU Medical	100°	670	185	7.4	8.6	3.6	12	275°	400 × 400	2–50
Uscope 3033A, Pusen Medical Technology Co.	120°	650	220	7.5	7.5	3.6	3	270°	400 × 400	3–50
Uscope 3022/3022A Pusen Medical Technology Co.	90°	650	220	9.2	9.2	3.6	3	270°	250 × 250	3–50
RP-U-C12, Redpine	85°	670	-	9.12	8.7	3.6	3–9	275°	400 × 400	3–50
Urofino UV-US110-H, Seegen	110°	750	-	7.5	7.5	3.6	3	275°	400 × 400	5–50
Urofino UV-US100-H, Seegen	110°	750	-	9	9	3.6	3	275°	400 × 400	5–50
LScope URS3006E, Seplou Medical	120°	650	-	6.6	7.5	3.6	9	275°	400 × 400	3–50
LScope URS3016E, Seplou Medical	120°	650	-	7.2	9	3.6	9	275°	400 × 400	3–50
FLEX-XC1, Storz	105°	700	-	9	9	3.5	3	270°	400 × 400	5–50

* FoV: Field of View.

**Table 2:** Unique features of currently available digital su-fURSs.

	Lightest Weight	Smallest Tip Diameter	Smallest Outer Shaft Diameter	Smallest Working Channel	Widest Deflection
FLEX-XC1, Storz				X	
Uscope 3033A, Pusen Medical Technology Co.			X		
Urofino UV-US110-H, Seegen			X		
LScope URS3006E, Seplou Medical		X	X		
Neo-Flex NFU 9, Neoscope Inc.	X				
Axis Leo K1046369, Dornier MedTech			X		
HU-30S, HugeMed			X		X
HU-30, HugeMed					X

## Data Availability

No new data were created or analyzed in this study. Data sharing is not applicable to this article.
